# Thyroid Function and Cognitive Health: Rethinking the Relationship between ‘Suboptimal’ TSH/FT4 Levels and Cognitive Decline

**DOI:** 10.1002/alz70860_103988

**Published:** 2025-12-23

**Authors:** Dvir Dori, Bruna Seixas Lima, Carlos Tyler Roncero, Durjoy Lahiri, Michael J Borrie, Howard Chertkow

**Affiliations:** ^1^ Rotman Research Institute‐Baycrest, TORONTO, ON, Canada; ^2^ Rotman Research Institute, Toronto, ON, Canada; ^3^ Division of Neurology, Kingston Health Sciences Center, Kingston, ON, Canada; ^4^ Division of Geriatric Medicine, Western University, London, ON, Canada; ^5^ Parkwood Institute, London, ON, Canada; ^6^ Baycrest Academy for Research and Education, Toronto, ON, Canada; ^7^ Rotman Research Institute, Baycrest Health Sciences, Toronto, ON, Canada

## Abstract

**Background:**

Emerging hypotheses suggest that 'suboptimal' thyroid function, defined as higher‐normal TSH (2.0–4.5 µIU/ml) or low‐normal FT4, may increase the risk of cognitive decline and dementia.

**Method:**

This study tested these claims using data from 991 participants in the COMPASS‐ND cohort, aged 50–91, with cognitive statuses ranging from unimpaired to dementia. Thyroid function was classified as “optimal” (TSH<2.0 µIU/ml or FT4≥16.74 pmol/L) or “suboptimal.”

**Result:**

Chi‐square analysis of 883 participants (523 with FT4 data) found no significant association between “suboptimal” thyroid function and cognitive impairment (TSH: *χ* ^2^=0.1585, *p* = 0.69; FT4: *χ* ^2^=0.0027, *p* = 0.96). True hypothyroidism was rare, and 98.6% of participants with elevated TSH had normal FT4 levels.

**Conclusion:**

These findings refute claims that higher‐normal TSH or low‐normal FT4 increases cognitive decline risk, highlighting the need for caution in redefining TSH cutoffs or pursuing unproven thyroid supplementation as a cognitive health intervention.

